# Supplementary material for: Nanobody-thioesterase chimeras to specifically target protein palmitoylation
Source: Nat Commun. 2025 Feb 7;16:1445. doi: 10.1038/s41467-025-56716-x (PMC11805987; doi:10.1038/s41467-025-56716-x)
Supplement: Supplementary file 1 — Supplementary Information [file 41467_2025_56716_MOESM1_ESM.pdf]

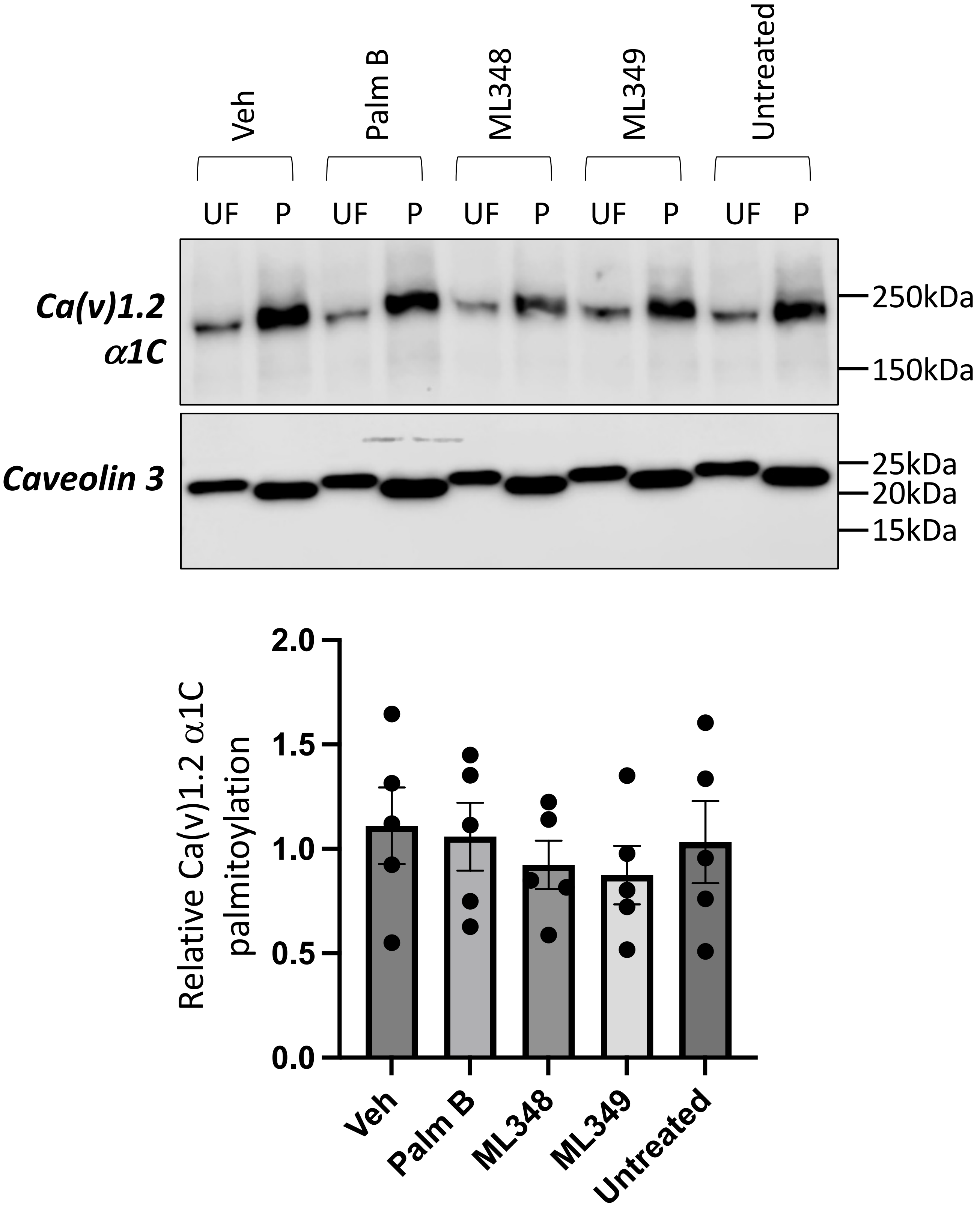

Supplementary Figure 1: Ca(v)1.2 α1C is not a substrate for APT1 or APT2. Adult rabbit ventricular myocytes were treated for 3 hours with inhibitors of APT1 (ML348, 10μM), APT2 (ML349, 10μM), the broad spectrum thioesterase inhibitor Palmostatin B (Palm B, 10μM) or vehicle (Veh, 0.1% DMSO). None of the thioesterase inhibitors investigated alter α1C palmitoylation, assessed using acyl-resin assisted capture and western blotting. UF: unfractionated cell lysate; P: purified palmitoylated proteins. Data are means ± SEM from n=5 independent experiments. Source data are provided as a Source Data file.

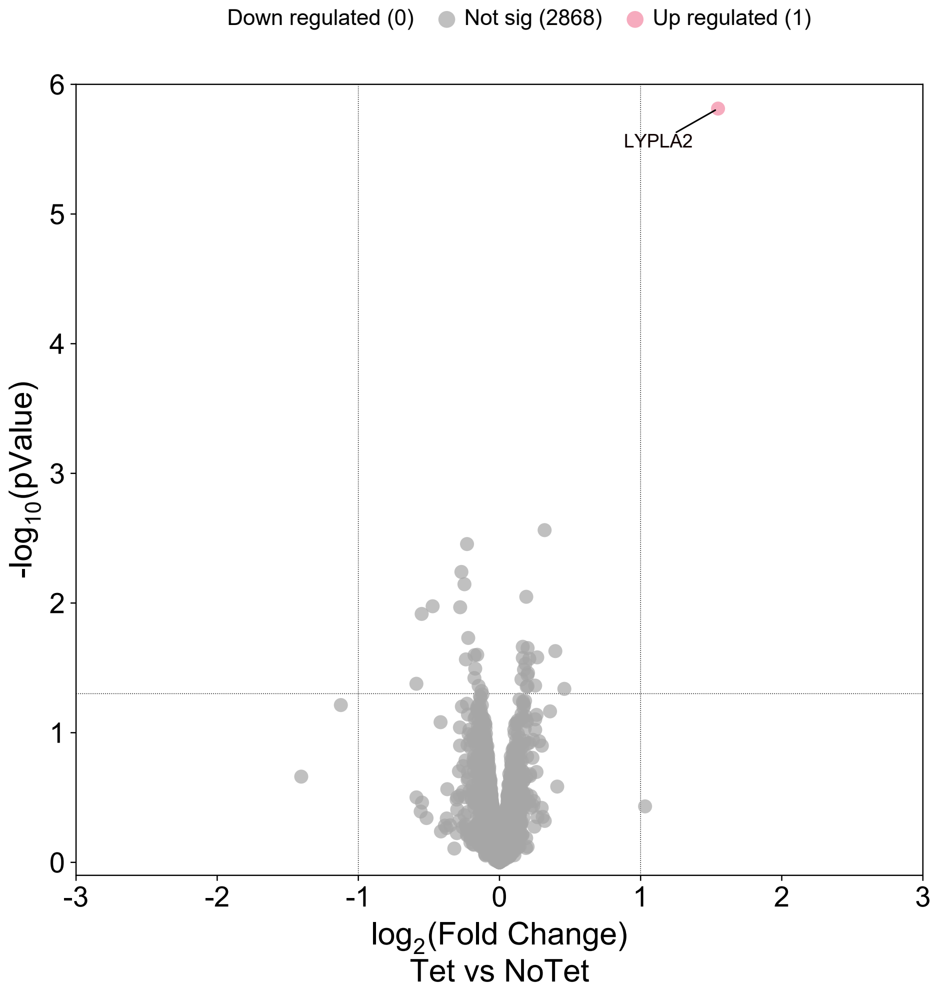

Supplementary Figure 2: Off-target activity of thioesterase nanobody chimeras assessed via quantitative proteomics. Whole proteome analysis of Flp-In T-REx cells stably expressing tetracycline-inducible APT2-G97-LAMA. Expression of a single protein is upregulated following induction of the nanobody chimera expression. Data are presented as log2 fold change values with a cut-off of 0.58 and a q-value threshold of 0.05. Statistical comparisons: moderated, two-sided t tests with adjustments for multiple comparisons using the Benjamini-Hochberg procedure. Data from n=3 independent experiments.

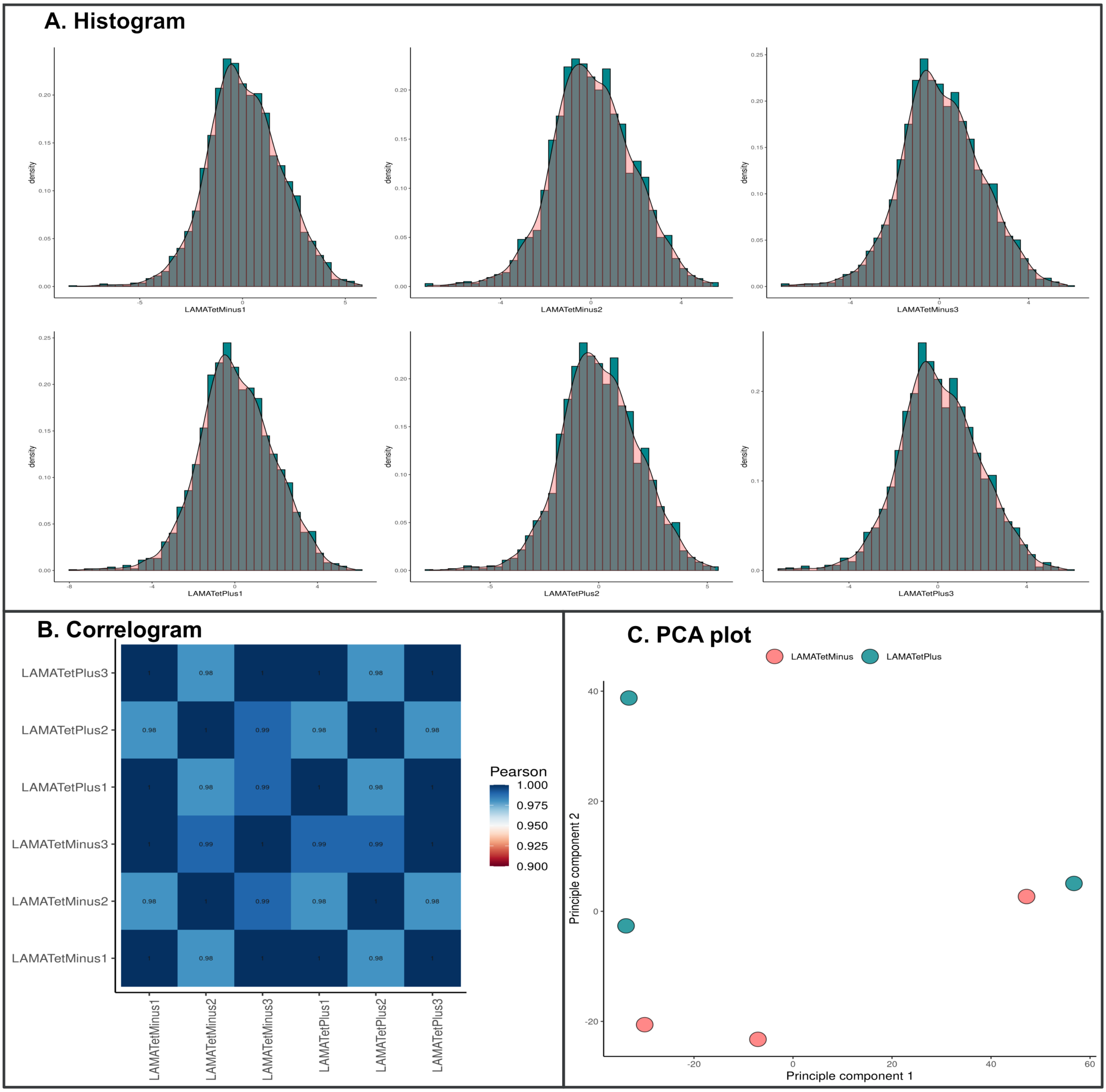

Supplementary Figure 3: Quality metrics for the global proteome analysis. A: Histogram for each of sample shows normal distribution of intensity of proteins. **B:** Correlogram shows that all the samples correlated with Pearson correlation coefficient >0.97. **C:** PCA plot illustrates no significant difference between tet minus (no expression of APT2-LAMA-G97) and tet plus (APT2 LAMA-G97 expressed) samples.

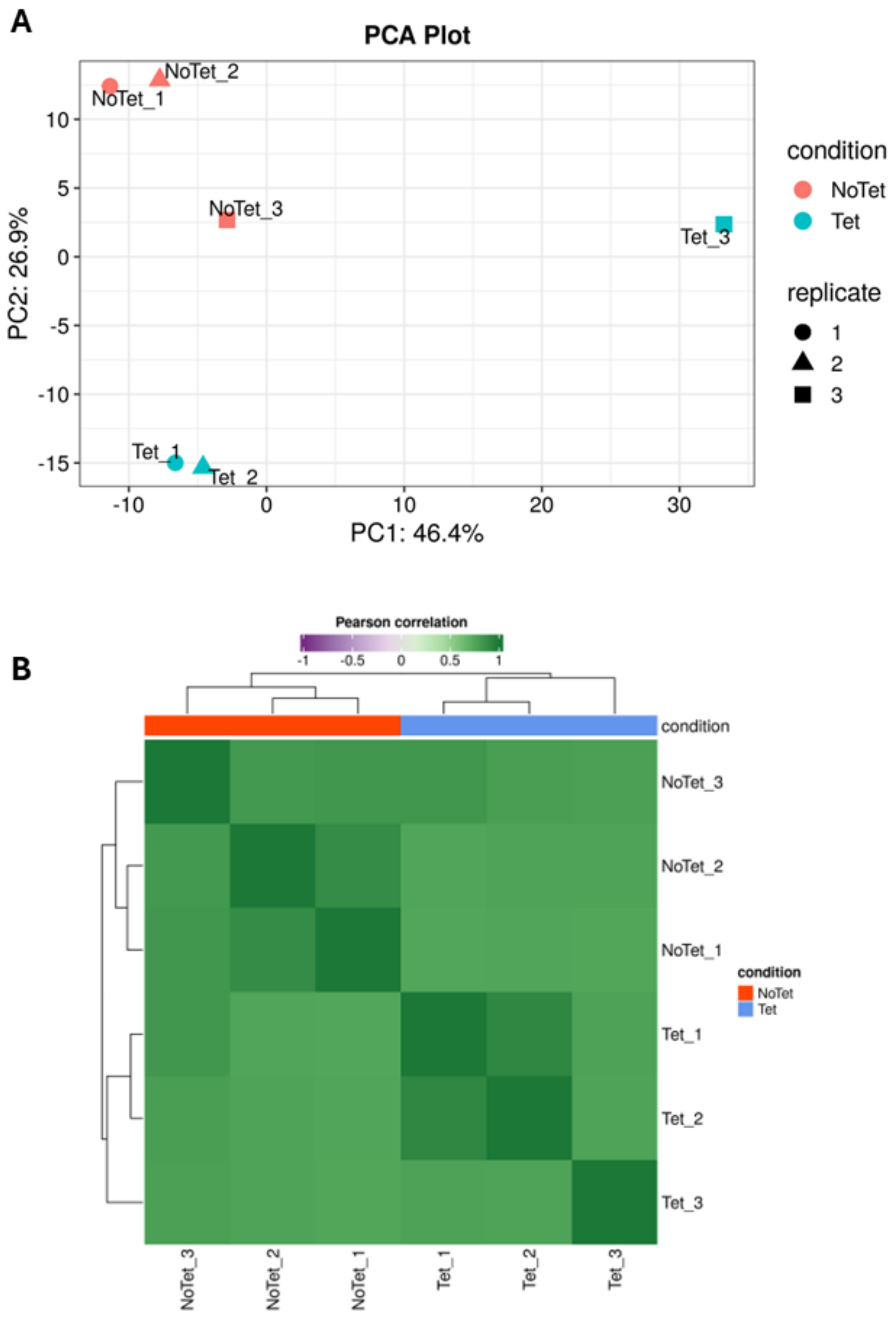

Supplementary Figure 4: Quality metrics for the palmitoylated proteome analysis: A: PCA plot shows differences between the palmitoyl proteome between NoTet (no expression of APT2-LAMA-G97) and Tet (APT2 LAMA-G97 expressed) samples. **B:** Correlation plot between replicates shows Pearson correlation coefficient of more than 0.85.

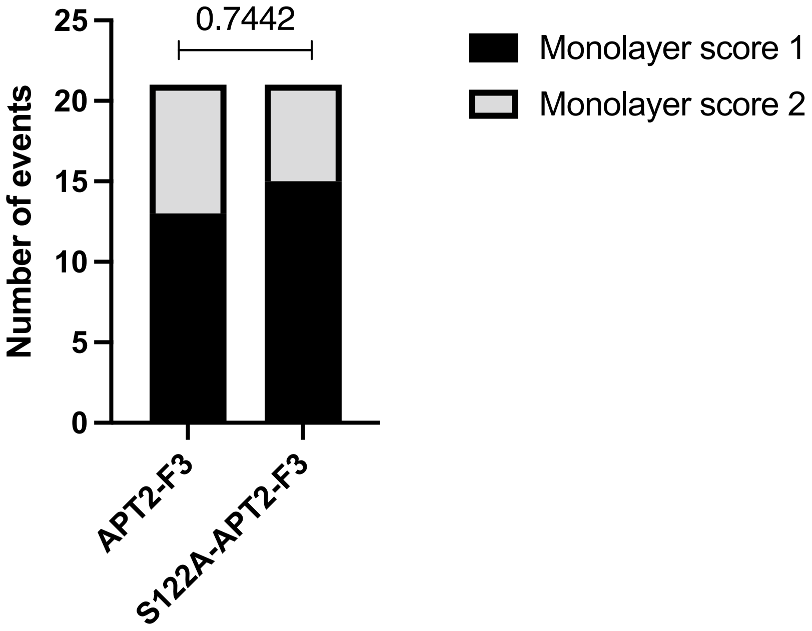

Supplementary Figure 5: iPSC-CM monolayer scores. iPSC-CMs were transfected with APT2-F3 or catalytically inactive (S122A) APT2-F3 and monolayer integrity assessed 48 hours later. Monolayer scores: 0: intact monolayer, 1: some small holes appearing, 2: larger and more frequent holes, 3: peeling/cell death. Statistical comparison: Two-tailed Fisher's Exact Test (N=21). Source data are provided as a Source Data file.
