# Supplementary material for: Nanobody-thioesterase chimeras to specifically target protein palmitoylation
Source: Nat Commun. 2025 Feb 7;16:1445. doi: 10.1038/s41467-025-56716-x (PMC11805987; doi:10.1038/s41467-025-56716-x)
Supplement: Supplementary file 2 — Description of Additional Supplementary Information [file 41467_2025_56716_MOESM2_ESM.docx]

**Description of Additional Supplementary Files**

File Name: Supplementary Data 1

Description: Whole proteome and palmitoylated proteome in Flp-In T-REx cells engineered to express tetracycline inducible APT2-LAMA-G97. Data are presented as log2 fold change values with a cut-off of 0.58 and a qvalue threshold of 0.05. Statistical comparisons: moderated, two-sided t-tests with adjustments for multiple comparisons using the BenjaminiHochberg procedure. Data from n=3 independent experiments.

File Name: Supplementary Movie 1-3

Description: : Live cell imaging to monitor eGFP-Spry2 redistribution upon trimethoprim withdrawal from HEK293T cells expressing eGFP-Spry2 and APT2- LAMA-G97. The number if the upper left corner indicates the time (minutes) since trimethoprim withdrawal. Note that LAMA-G97 is based on the GFP enhancer nanobody. When trimethoprim is withdrawn to allow the nanobody to engage GFP, nanobody binding increases the fluorescent intensity of the GFP. The increase in eGFPSpry2 fluorescence intensity caused by the nanobody binding leads to some image saturation. Scale bar: 10 µm.
