# Supplementary material for: Nanobody-thioesterase chimeras to specifically target protein palmitoylation
Source: Nat Commun. 2025 Feb 7;16:1445. doi: 10.1038/s41467-025-56716-x (PMC11805987; doi:10.1038/s41467-025-56716-x)

1B

Ca(v)1.2 I-II linker (GFP)

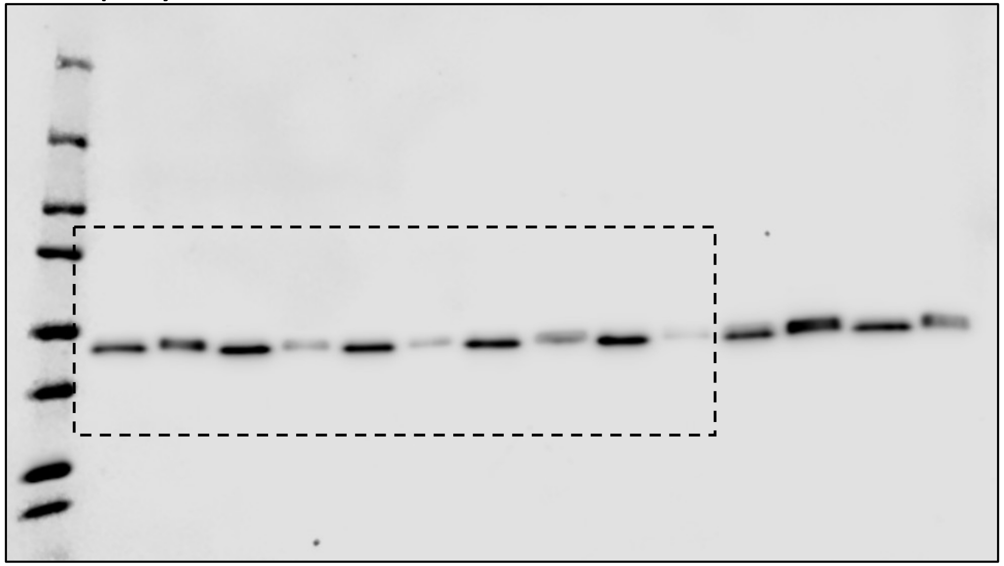

FLAG (Nanobody)

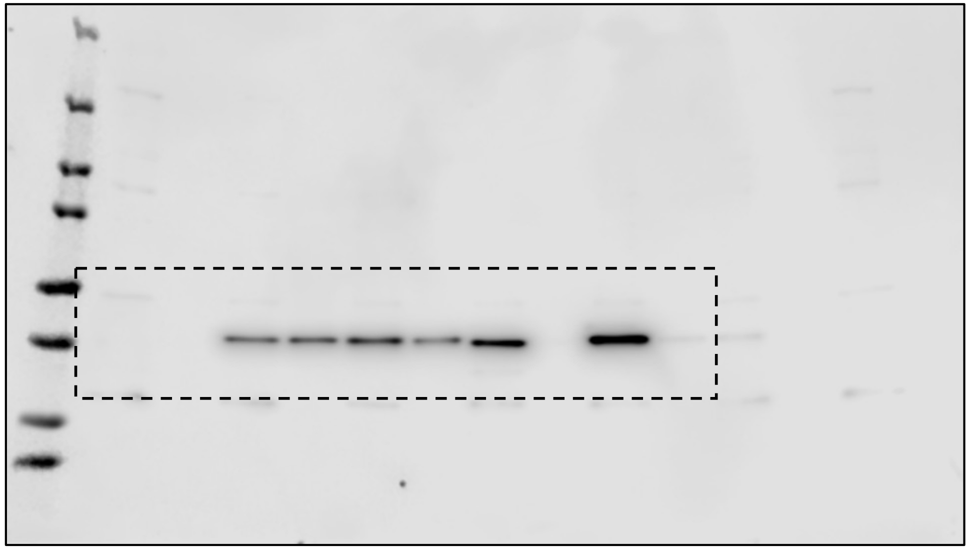

Flot2

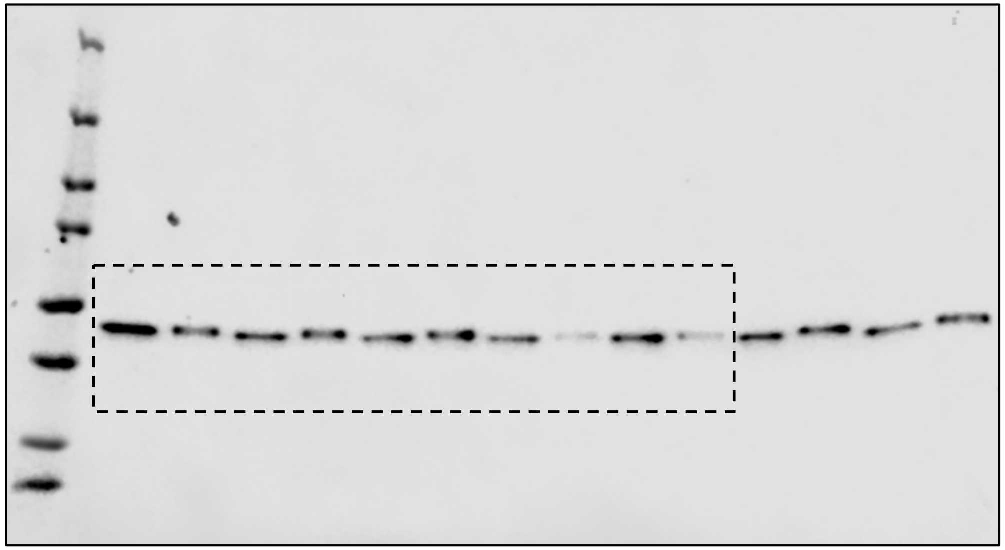

1C

Ca(v)1.2 I-II linker (GFP)

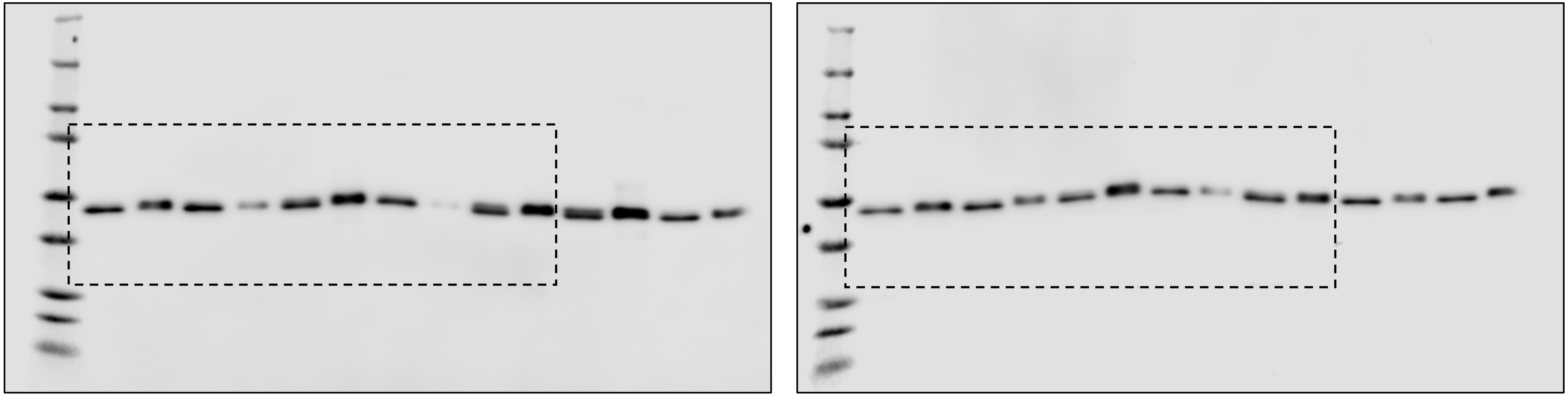

Flot2

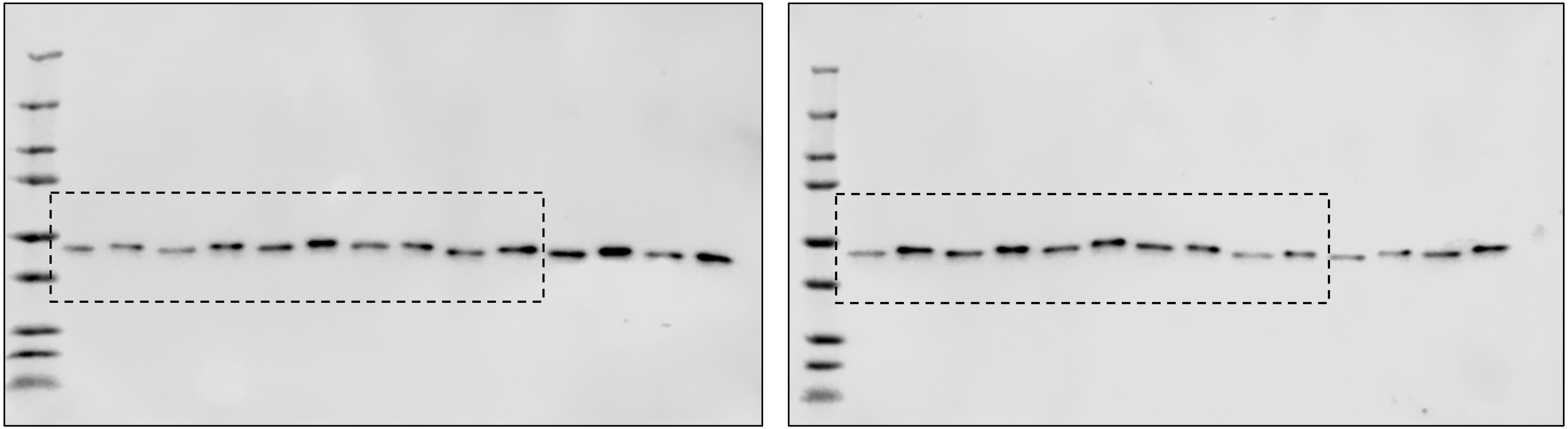

1C (cont)

FLAG (Nanobody)

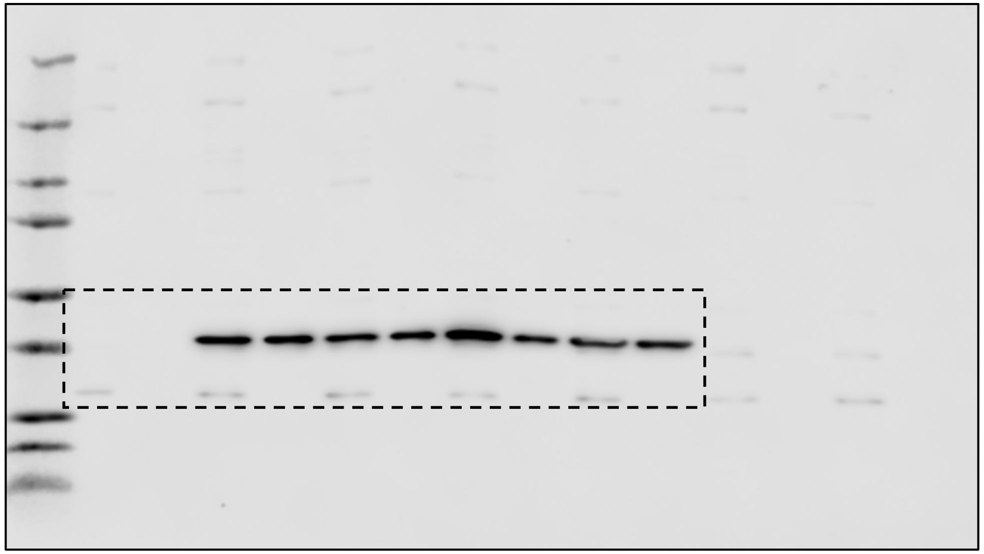

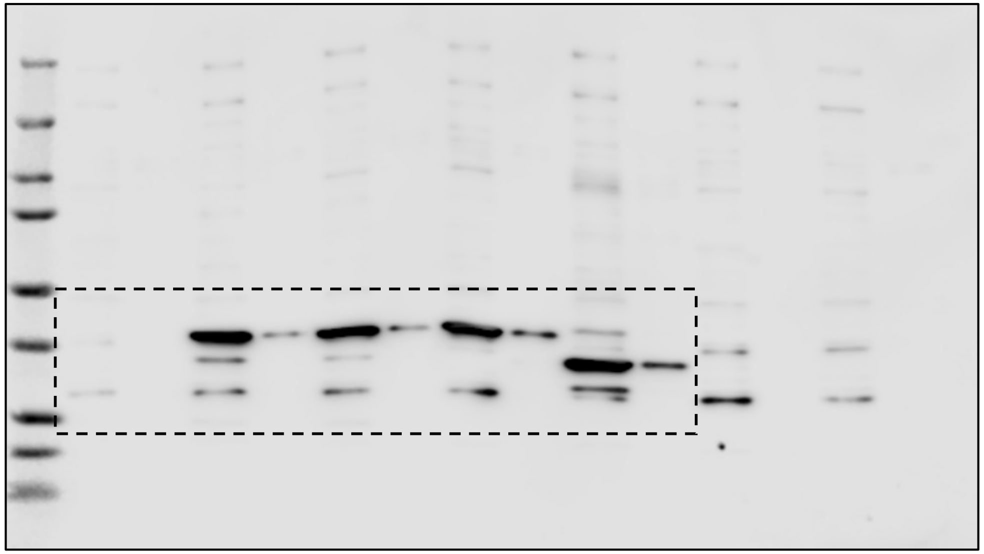

1E

FLAG
(Nanobody)

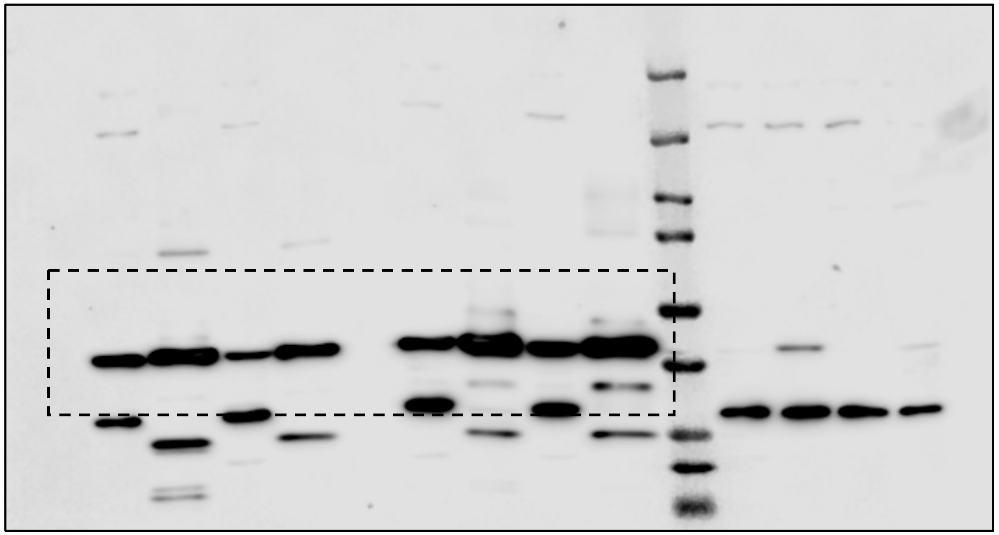

Ca(v)1.2 I-II
linker (GFP)

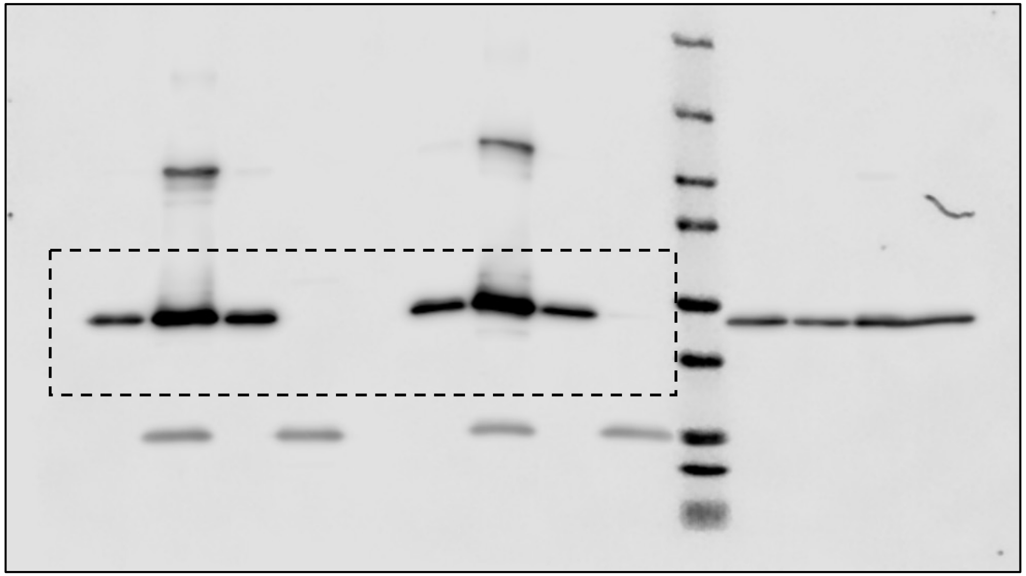

1F

Ca(v)1.2 I-II linker (GFP)

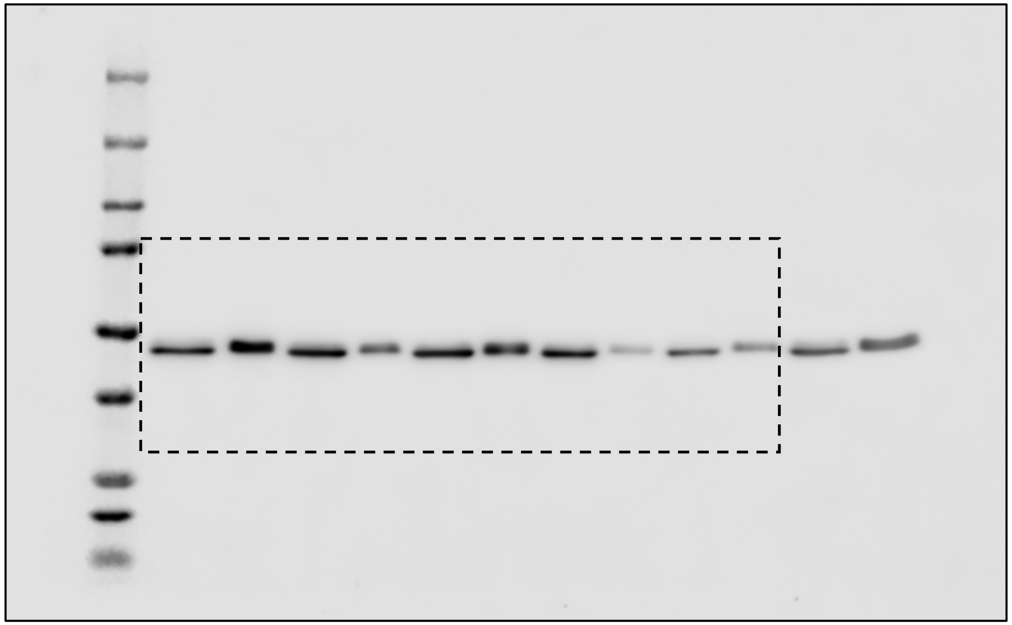

FLAG (Nanobody)

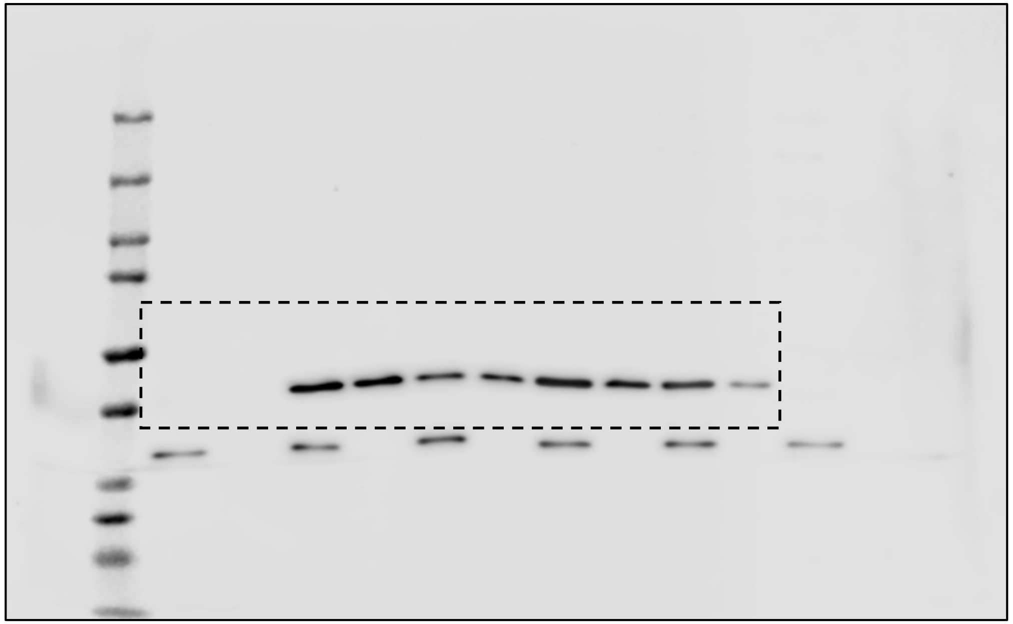

Flot2

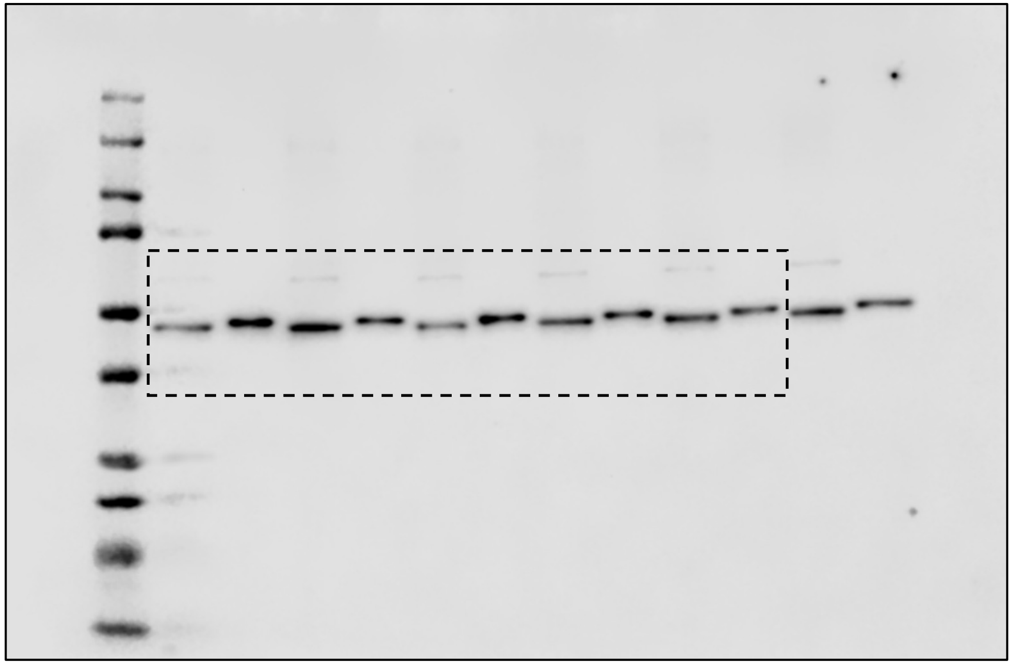

2A *PLM-YFP*

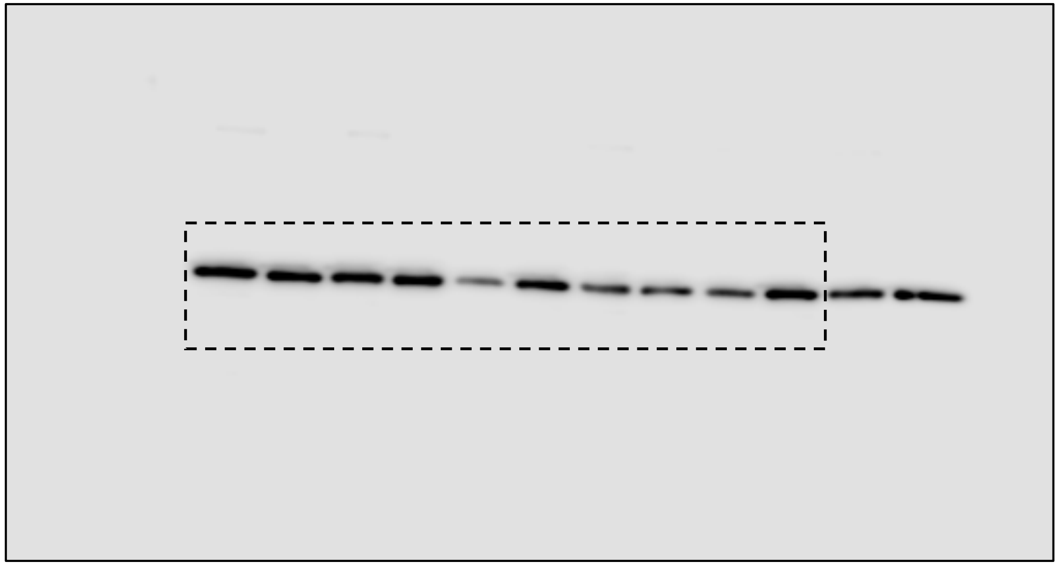

Flot2

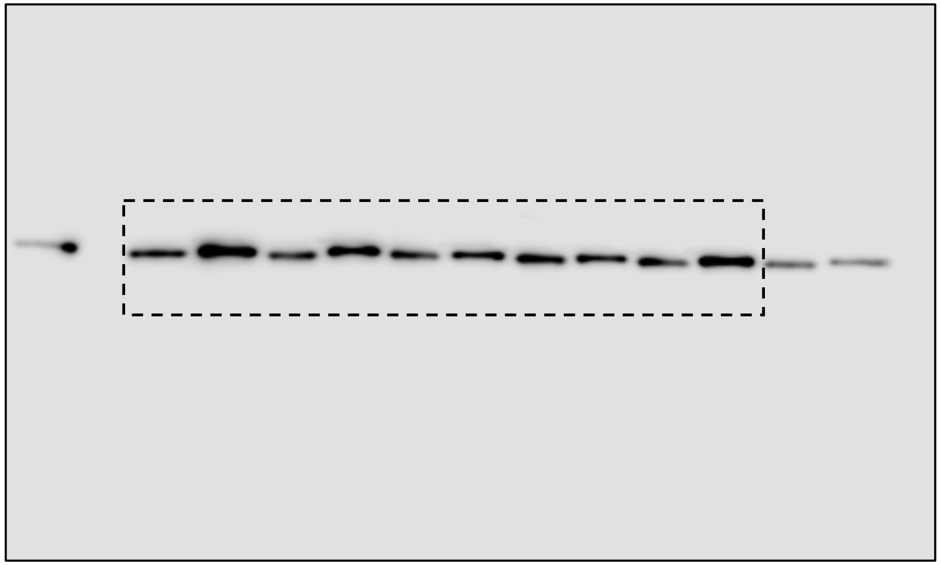

FLAG
(Nanobody)

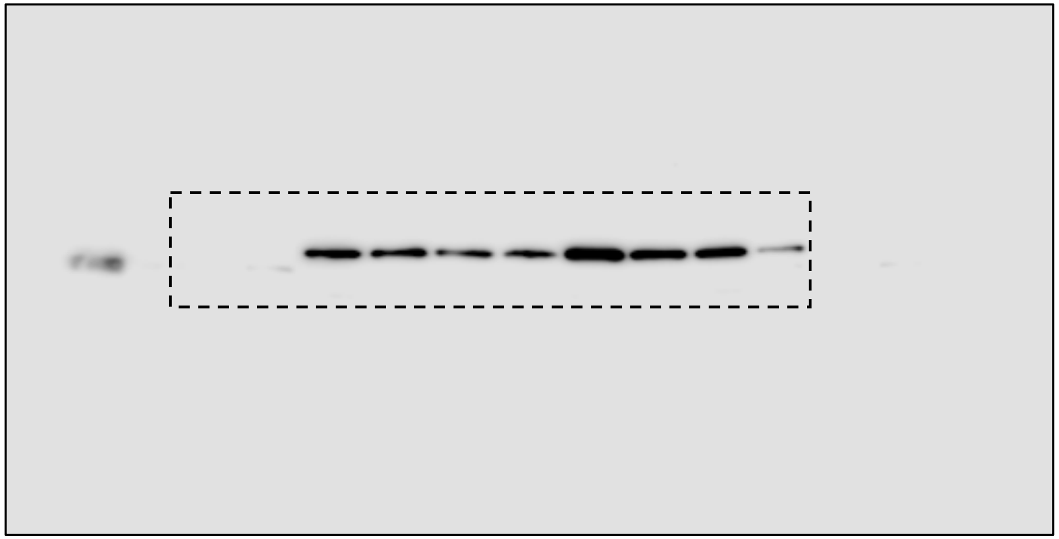

2B

NCX1-YFP

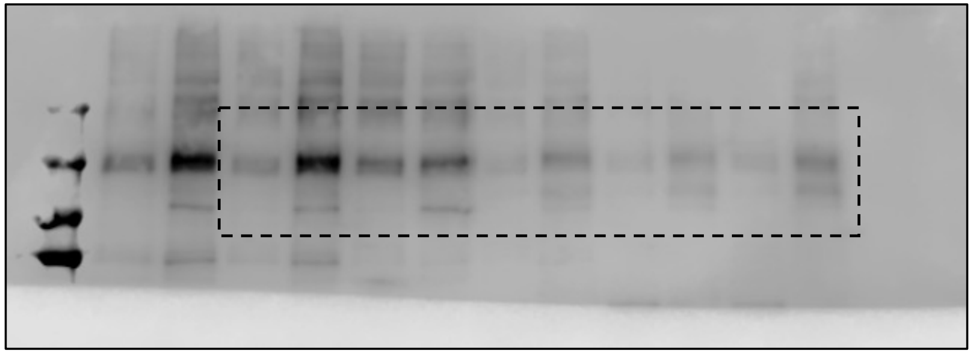

Flot2

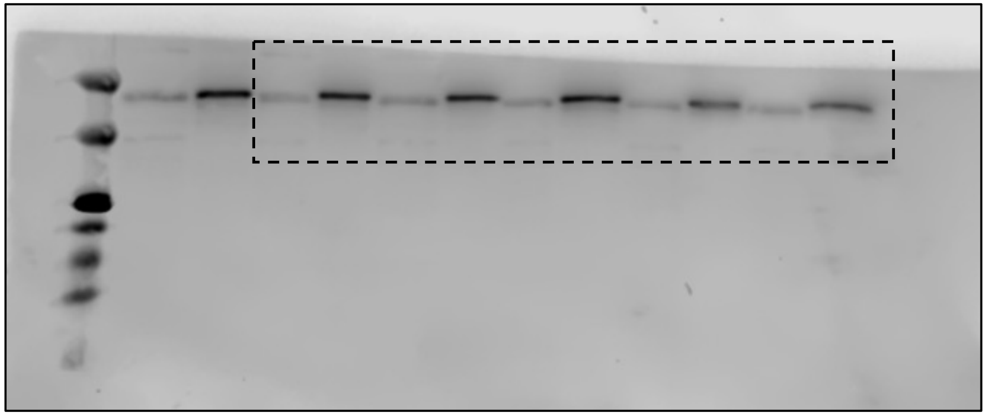

FLAG (Nanobody)

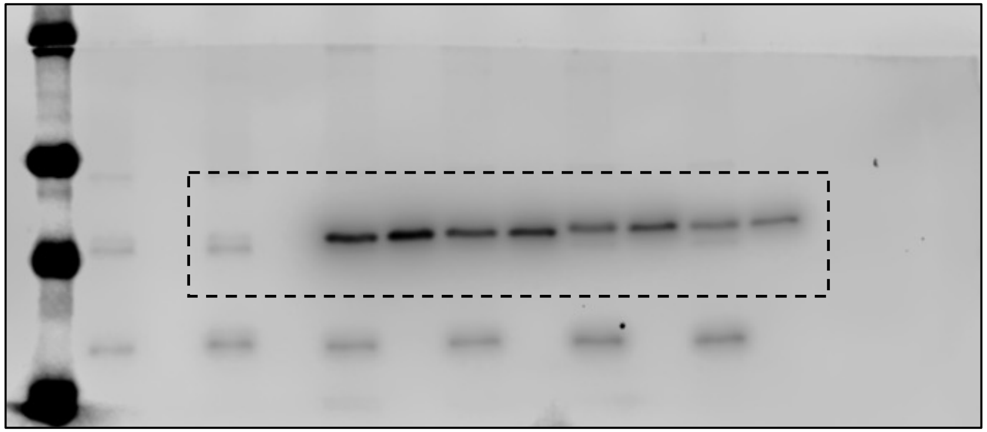

2C

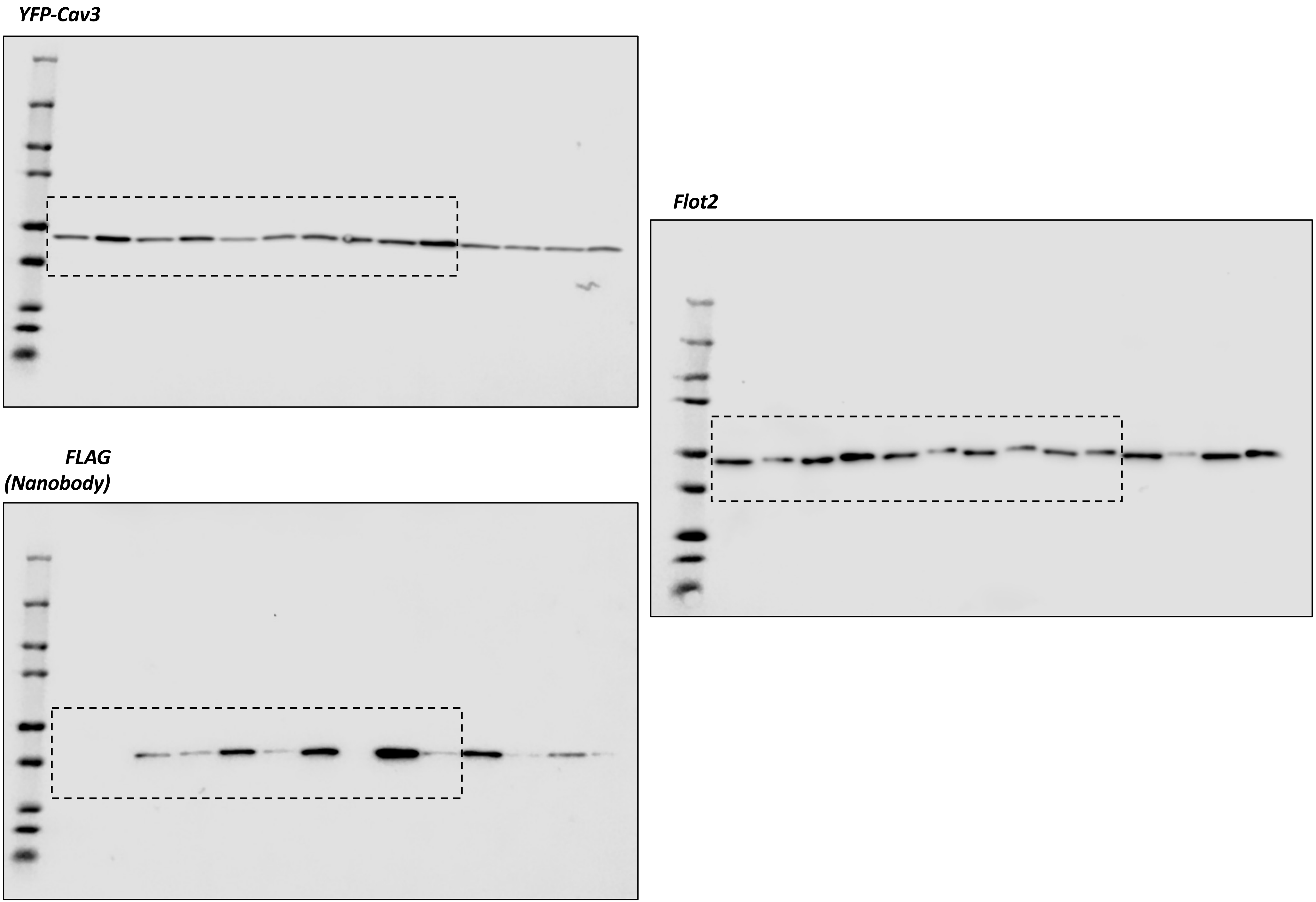

3B

Ca(v)1.2 I-II linker

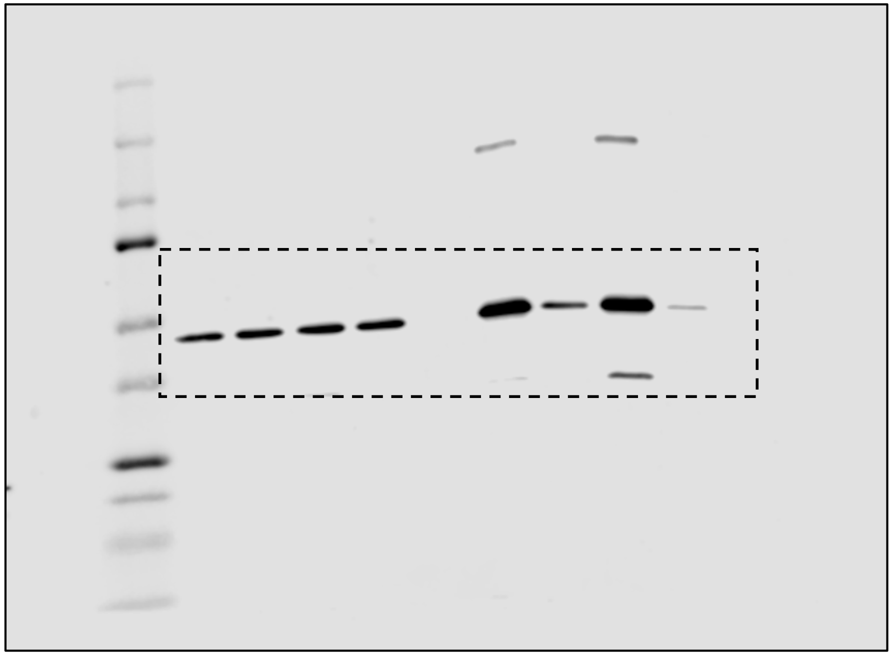

FLAG (Nanobody)

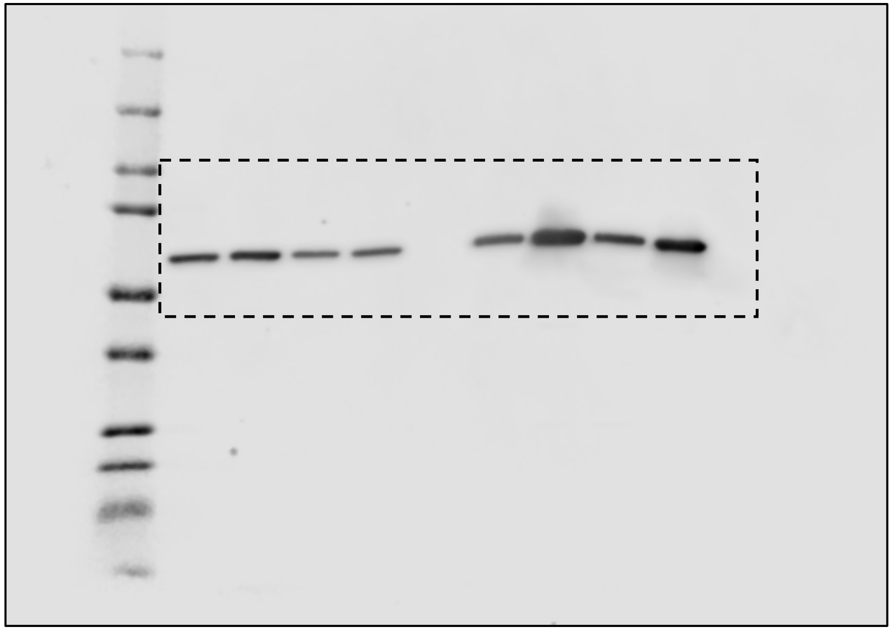

3C

Ca(v)1.2 I-II linker

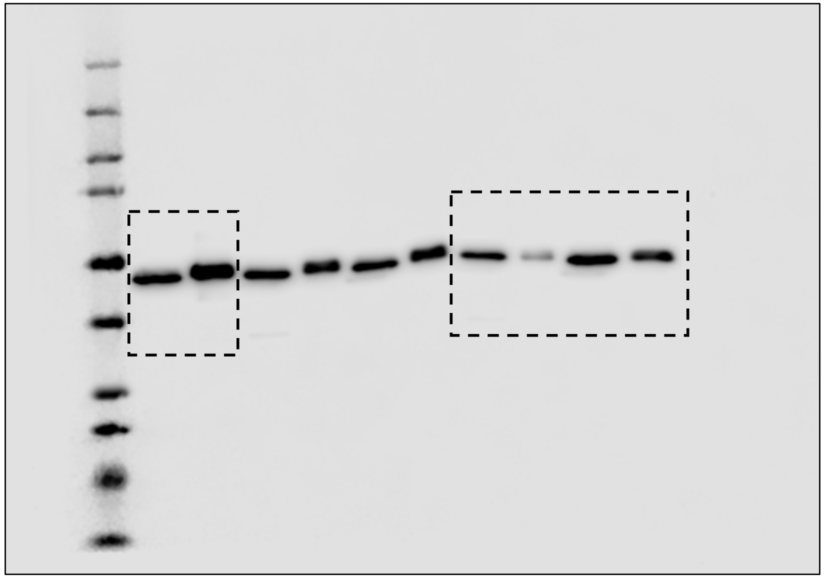

FLAG (Nanobody)

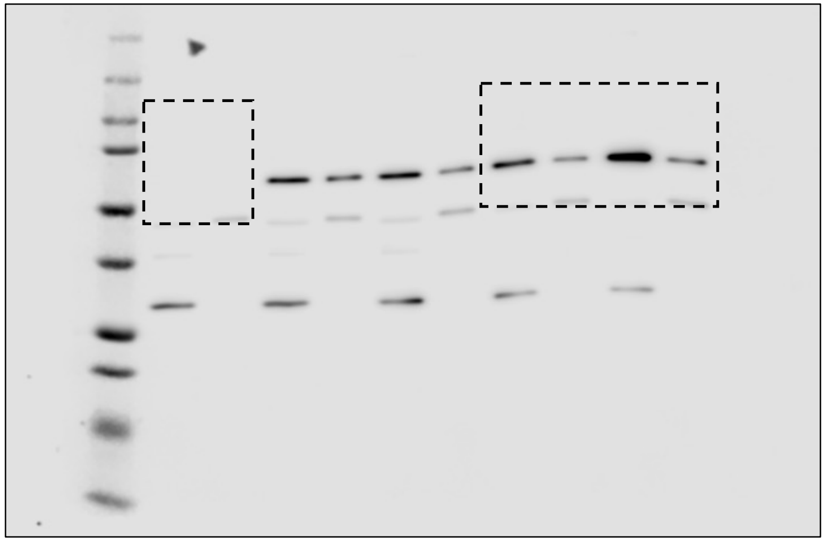

Flot2

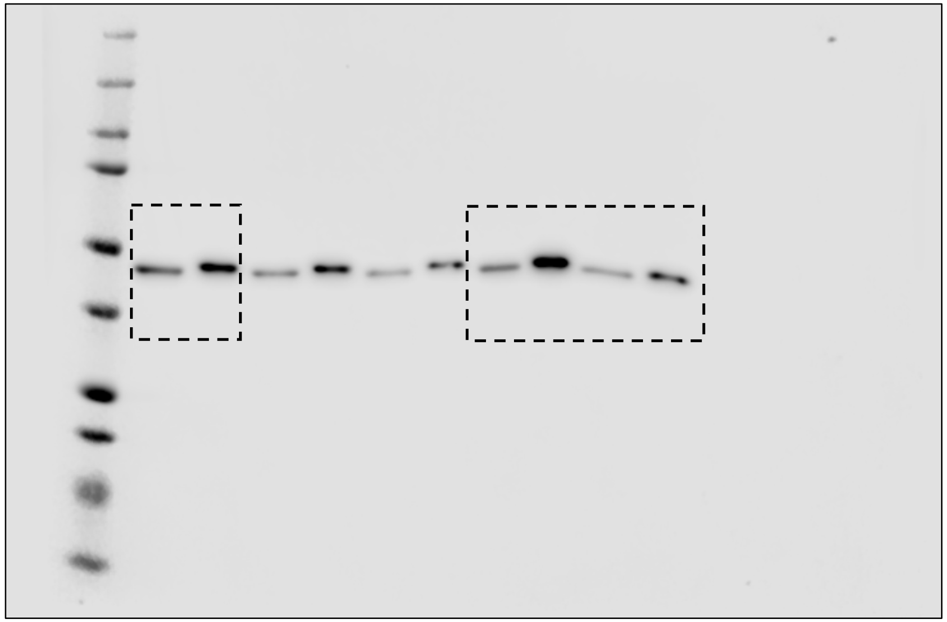

3D

Ca(v)1.2 I-II linker

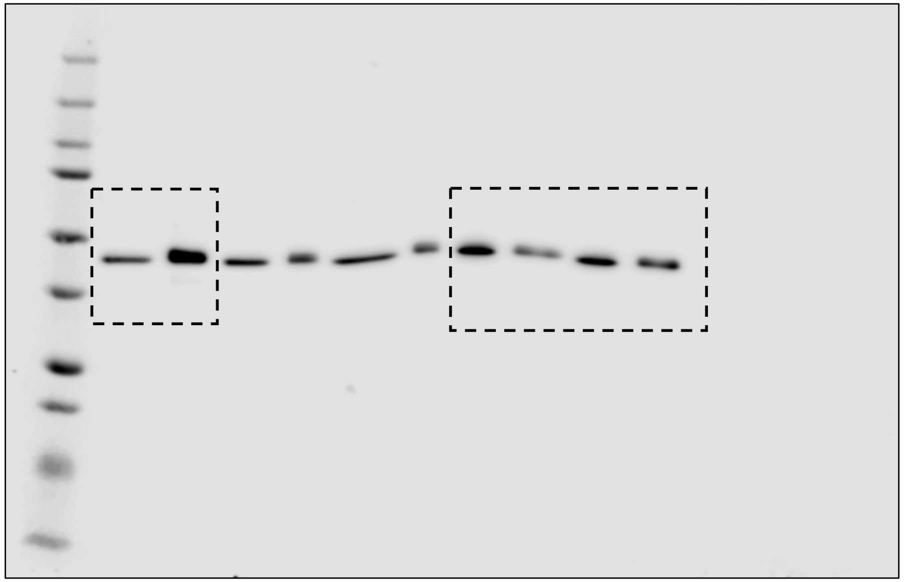

Flot2

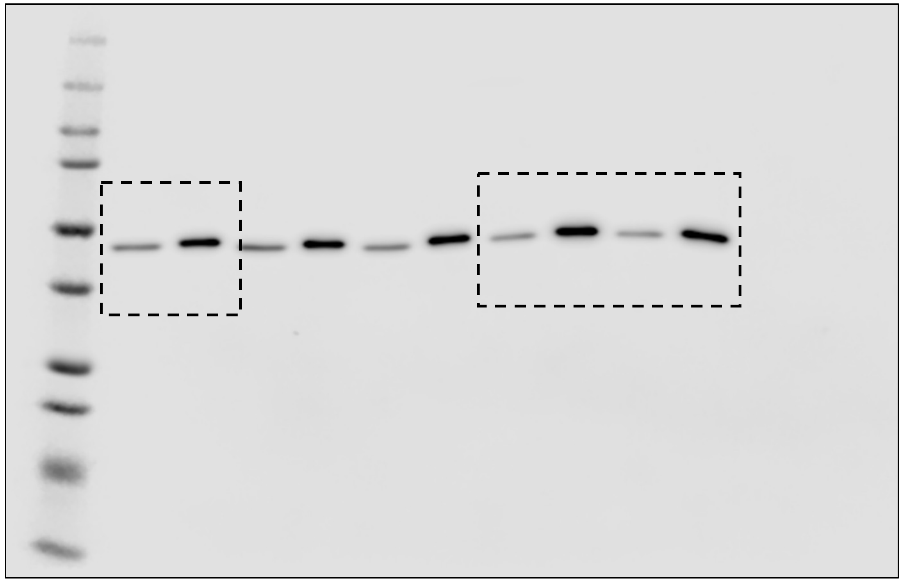

FLAG (Nanobody)

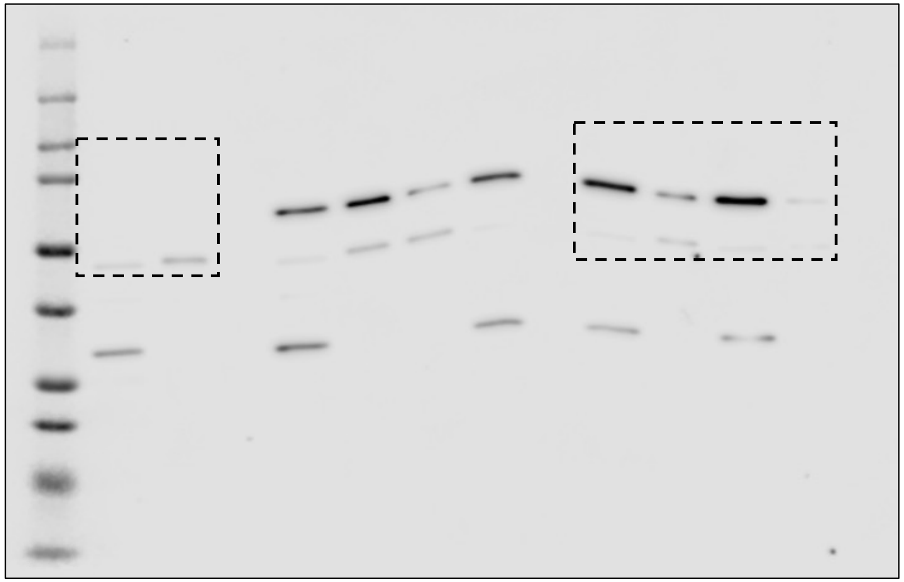

4A *GFP-Spry2*

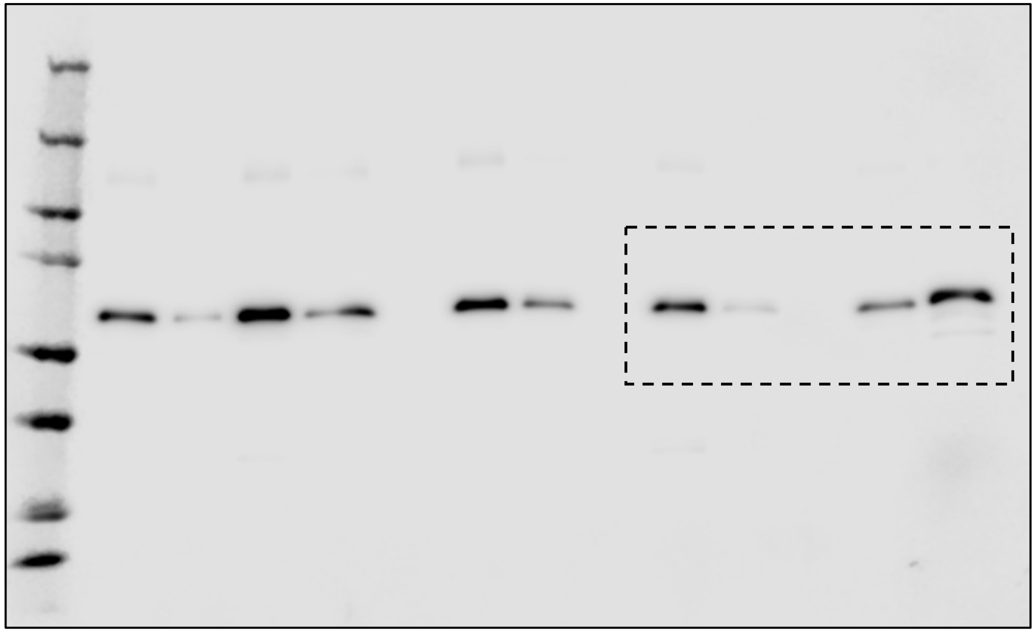

FLAG (Nanobody)

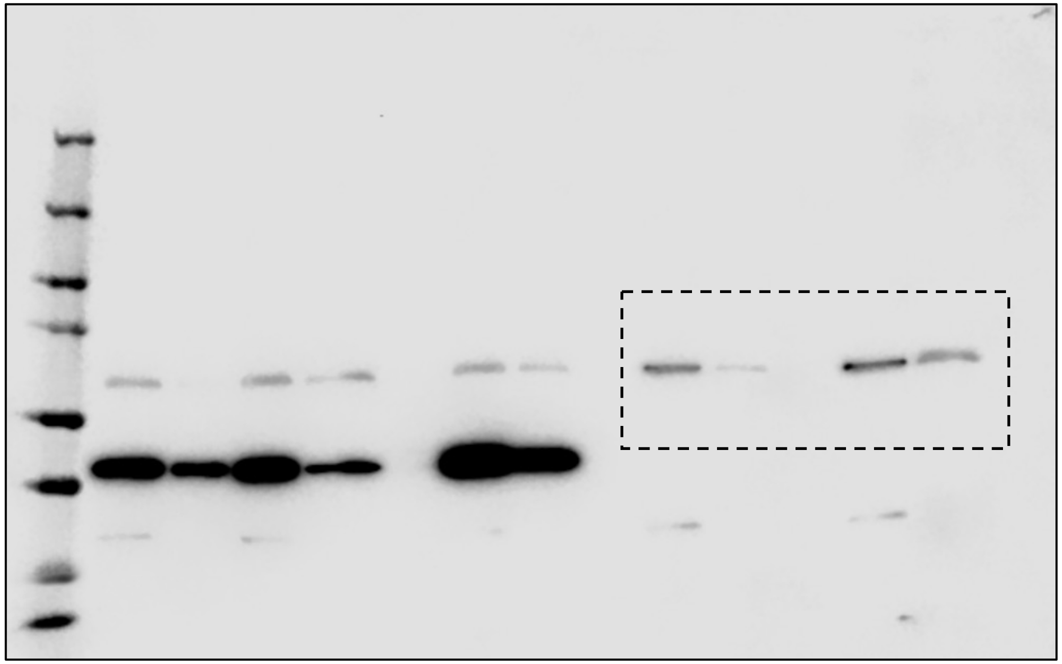

Flot2

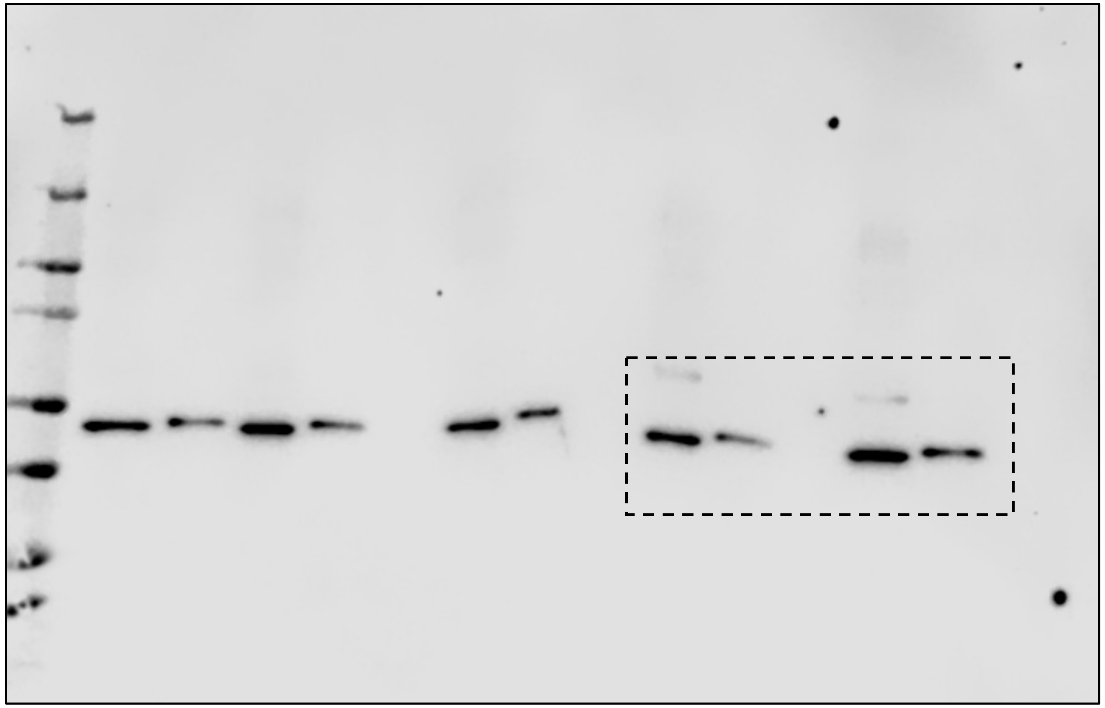

4B

GFP-Spry2

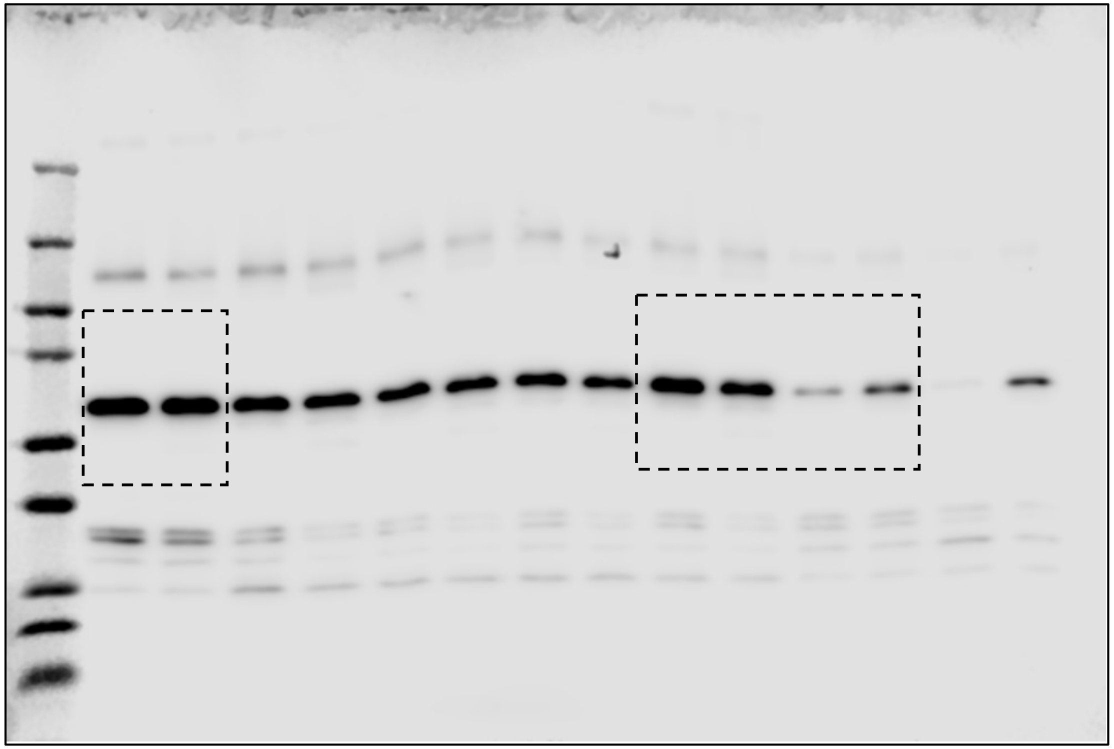

GAPDH

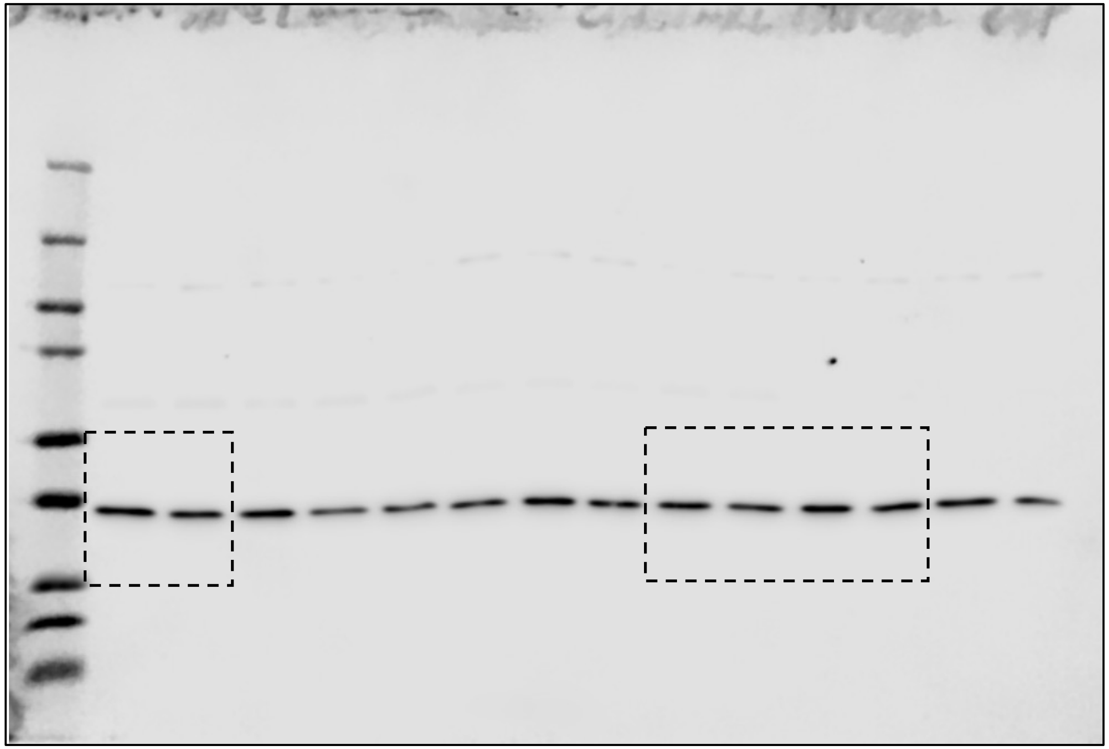

5A

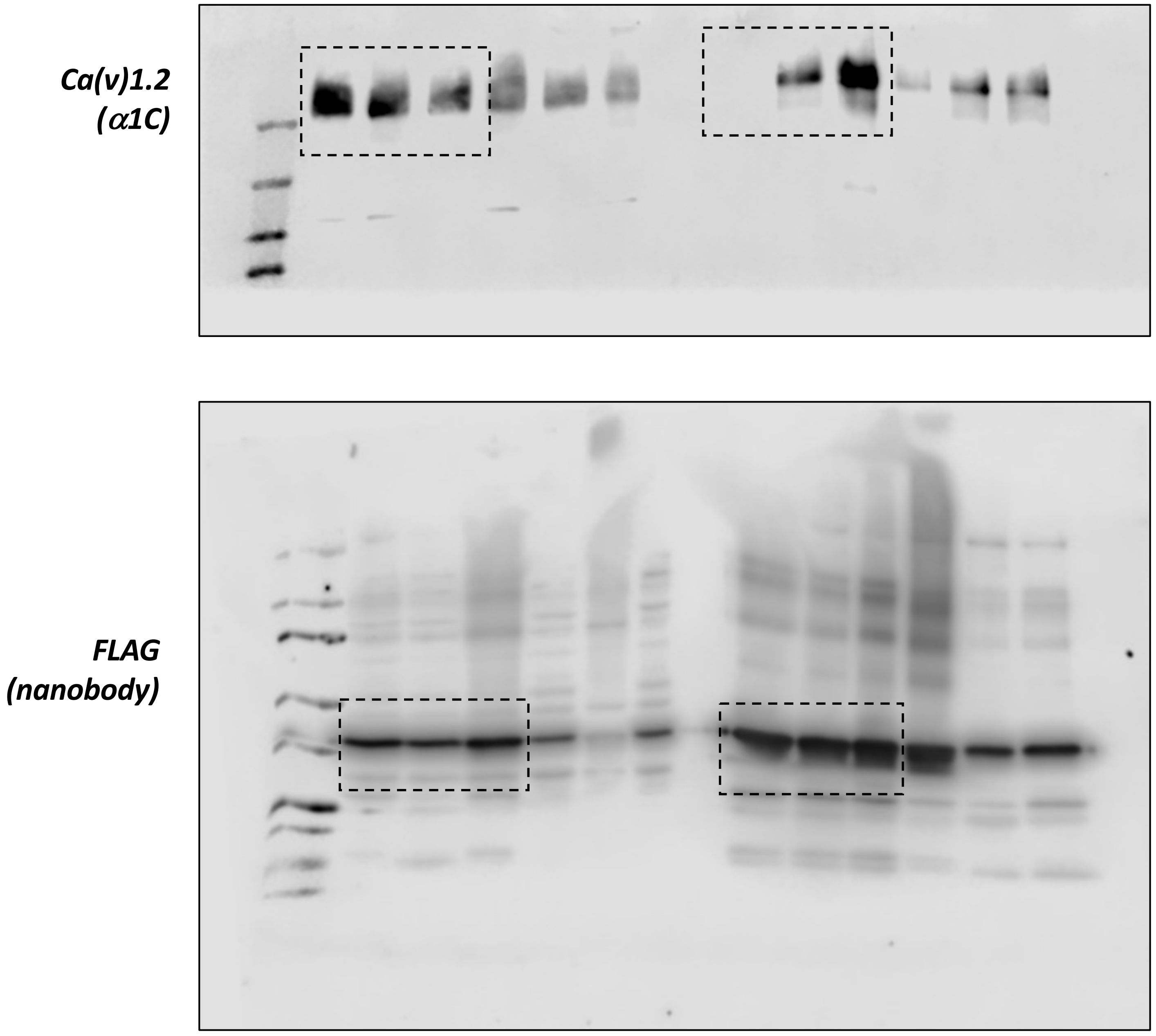

5A cont

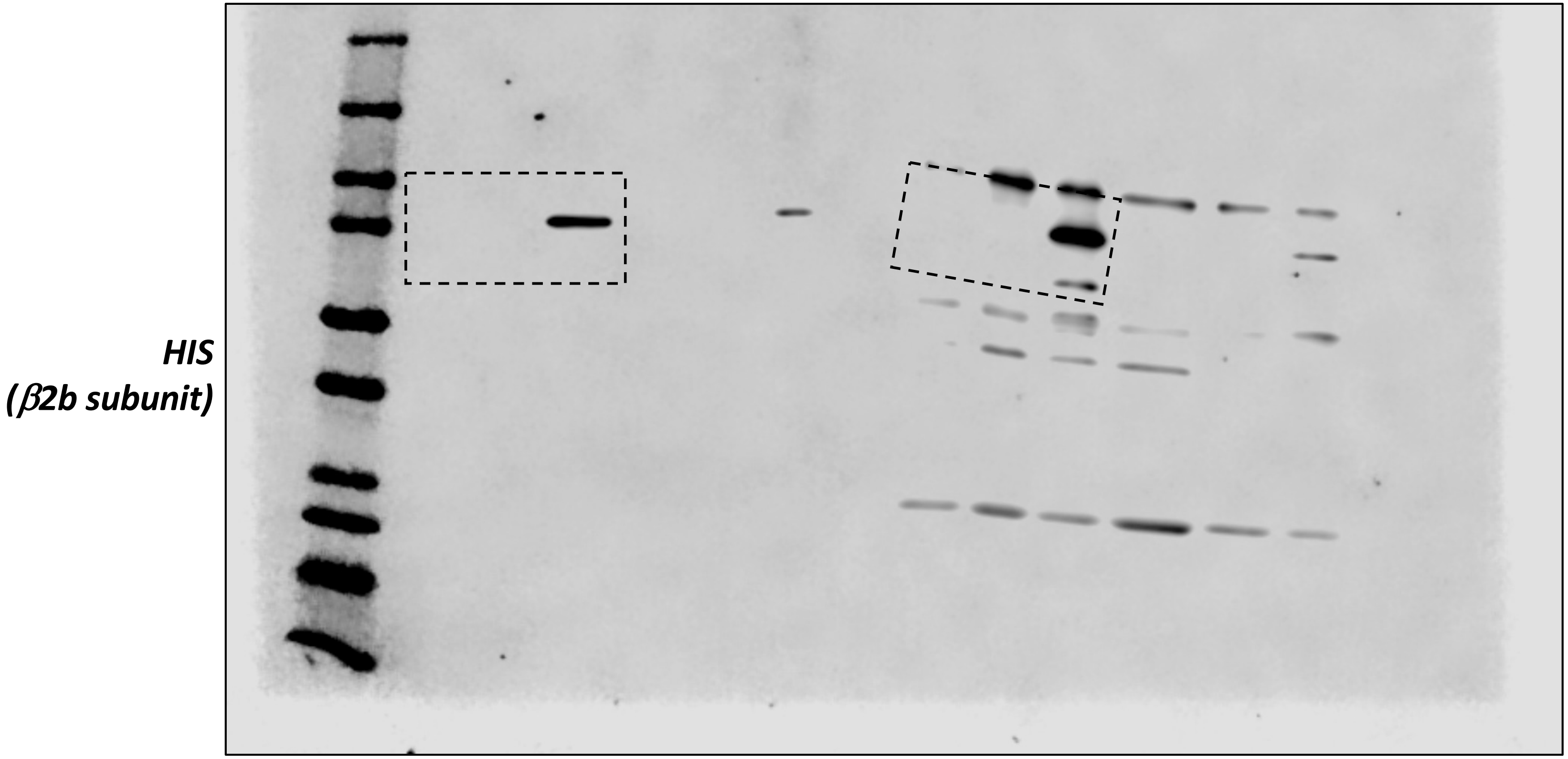

5A cont

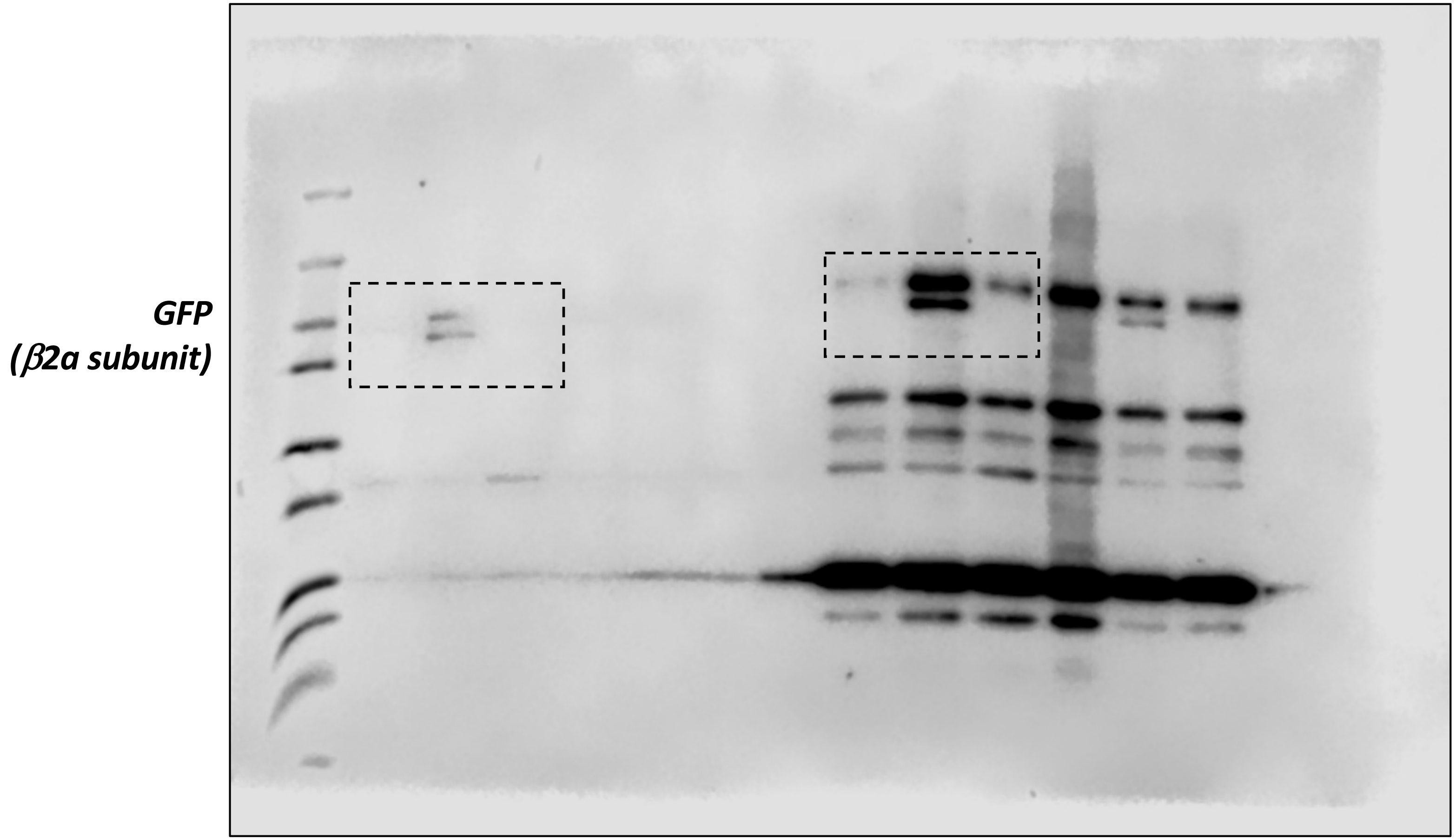

5B

Ca(v)1.2 (α1C)

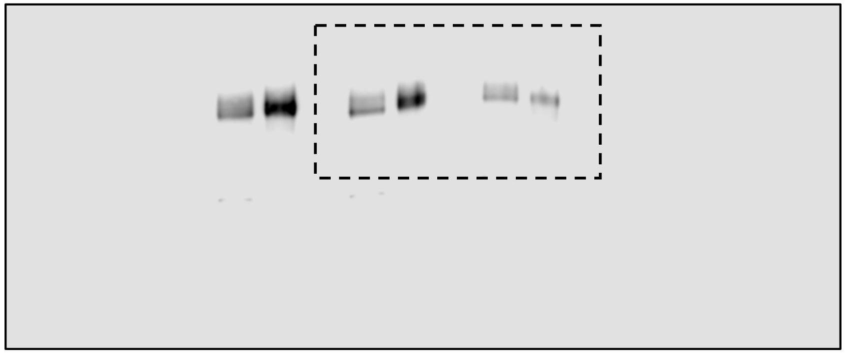

FLAG (nanobody)

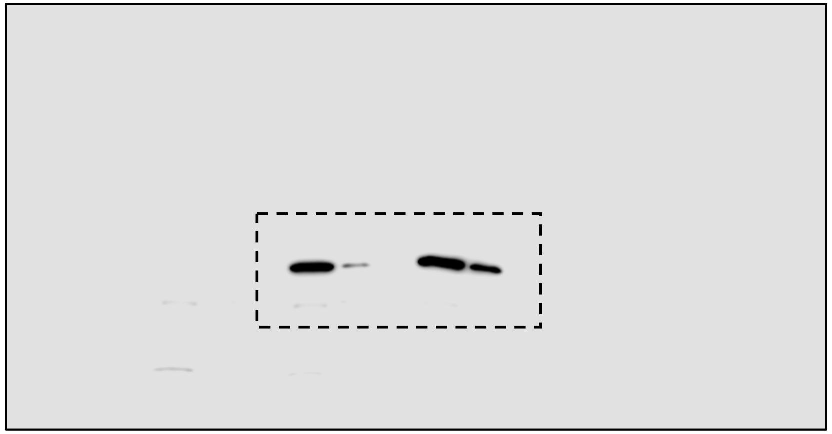

HIS (β2b subunit)

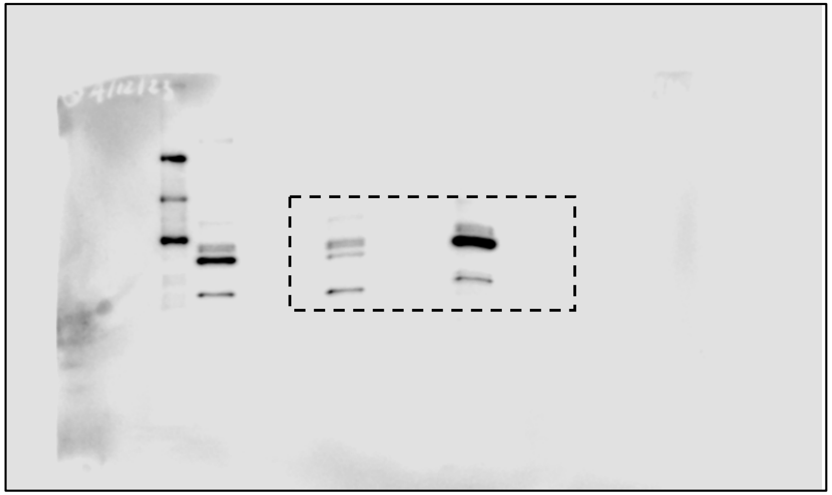

S1

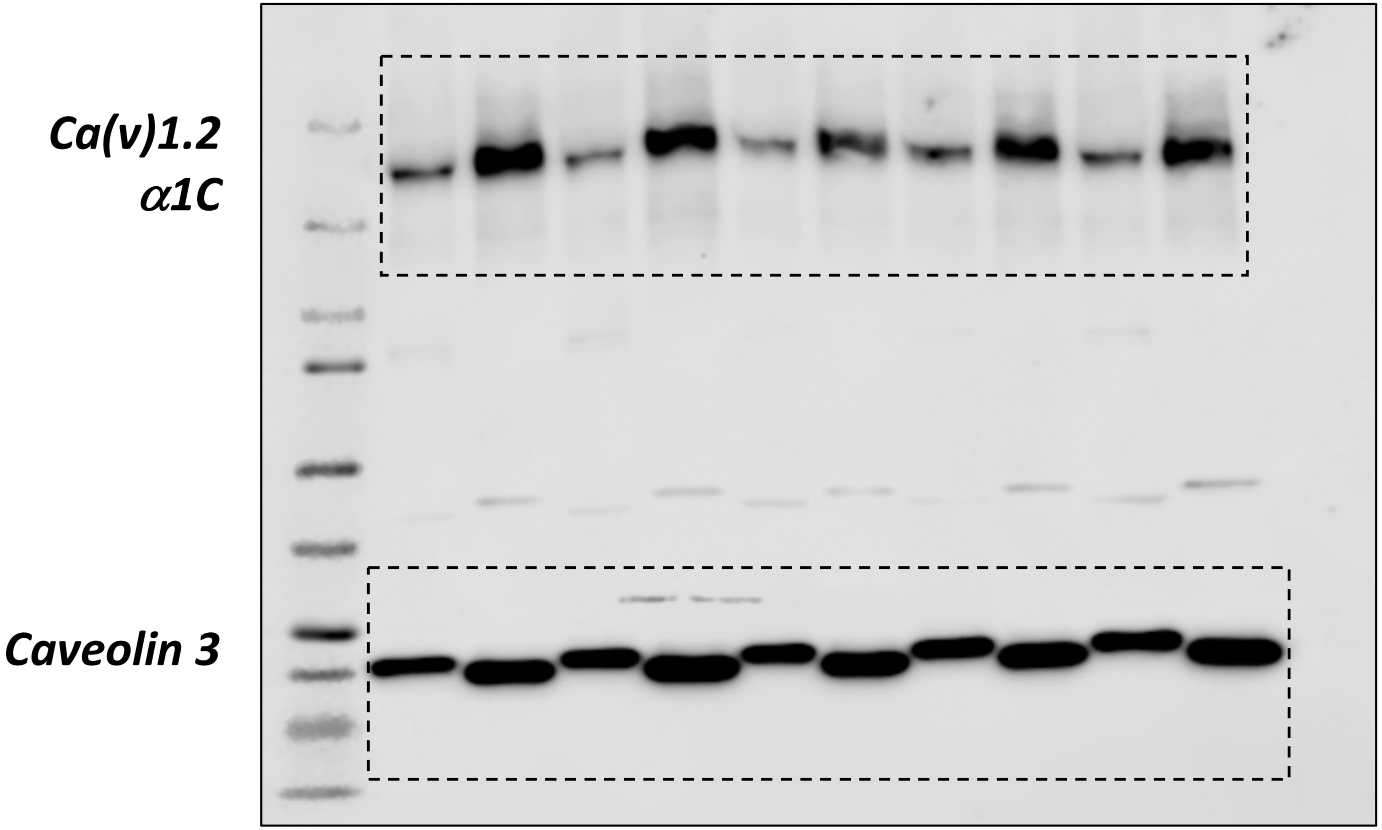

Supplement: Supplementary file 9 — Source Data [file 41467_2025_56716_MOESM9_ESM.zip › Source data/Uncropped blots.pdf]
